# Glycogenin-1 deficiency: a case report and review of the literature

**DOI:** 10.3389/fgene.2026.1777448

**Published:** 2026-05-29

**Authors:** Nicola Molitierno, Daniele Velardo, Giulia Salvucci, Elena Abati, Gabriele Tumminello, Michela Ripolone, Simona Zanotti, Laura Napoli, Patrizia Ciscato, Monica Sciacco, Giacomo Pietro Comi, Stefania Corti, Dario Ronchi

**Affiliations:** 1 Department of Pathophysiology and Transplantation, Dino Ferrari Center, University of Milan, Milan, Italy; 2 Neuromuscular and Rare Disease Unit, IRCCS Fondazione Ca' Granda Ospedale Maggiore Policlinico, Milan, Italy; 3 Neurology Unit, IRCCS Fondazione Ca' Granda Ospedale Maggiore Policlinico, Milan, Italy; 4 Department of Cardio-Thoracic-Vascular Diseases, IRCCS Fondazione Ca' Granda Ospedale Maggiore Policlinico, Milan, Italy

**Keywords:** case report, glycogen storage disease type XV, glycogenin-1, GSD XV, GYG1, PGBM2, polyglucosan bodies

## Abstract

Pathogenic biallelic variants in *GYG1*, encoding for glycogenin-1, are associated with polyglucosan bodies myopathy characterized by muscle accumulation of deposits of amylopectin-like polysaccharides (MIM 616199). So far, only few cases (<50) with molecular defects in *GYG1* have been reported. The proband is a 79-year-old Italian woman presenting with subacute onset of diffuse soreness, weakness in the upper limbs and diffuse muscle atrophy without cardiac or respiratory involvement. Electromyography showed myopathic features. Muscle biopsy revealed several type I muscle fibers containing intensely PAS-positive, diastase-resistant vacuoles of variable dimension. Ultrastructural analysis showed vacuoles with granular-fibrillar storage material localized in subsarcolemmal and intermyofibrillar areas, small amounts of free glycogen and jagged Z-line appearance of some sarcomeres. Clinical exome sequencing revealed two heterozygous pathogenic variants in *GYG1*. Our findings provide clinical and molecular characterization of a novel case of *GYG1*‐related polyglucosan bodies myopathy and highlight the histological clues leading to the diagnosis of this rare clinical phenotype.

## Introduction

Polyglucosan body myopathy type 2 (PGBM2, MIM 616199) is a rare metabolic disorder characterized by the abnormal accumulation of poorly branched, periodic acid–Schiff (PAS)-positive and variably diastase-resistant glycogen aggregates in skeletal muscle, known as polyglucosan bodies (PB) (Laforêt et al., 2017). These PBs are a hallmark of several rare glycogen storage and metabolic disorders, including glycogen branching enzyme (GBE1) deficiency, phosphofructokinase deficiency, adenosine monophosphate–activated protein kinase deficiency, RBCK1 (RanBP-type and C3HC4-type zinc finger–containing 1) deficiency, and Lafora disease, which results from mutations in *EPM2A* or *EPM2B (NHLRC1)*, encoding the interacting proteins laforin and malin (Hedberg-Oldfors and Oldfors, 2015).

PGBM2 is associated with mutations in the glycogenin-1 (*GYG1*) gene, which encodes glycogenin-1, a crucial protein for glycogen biosynthesis acting as the initial primer for glycogen chain formation. PGBM2 is classified as a subtype of glycogen storage disease type XV (GSD XV), all forms of which are caused by biallelic pathogenic variants in GYG1. The age of onset for this disorder varies widely, from childhood to the ninth decade of life. Though cardiac involvement, including cardiomyopathy and arrhythmia, has been reported in a subset of patients, most cases primarily affect the musculoskeletal system, typically manifesting as a slowly progressive, proximal, symmetrical weakness, although scapuloperoneal, distal, or asymmetric myopathy have been also reported (Malfatti et al., 2014; Ben Yaou et al., 2017; Stojkovic et al., 2018; Desikan et al., 2018; Akman et al., 2016; Lefeuvre et al., 2020). To date, fewer than 50 GSD XV/PGBM2 cases have been described, highlighting the rarity of this condition (Supplementary Table 1).

In this report, we present the case of a 79-year-old female patient with acute onset of progressive muscular weakness, ultimately diagnosed with PGBM2 associated with two heterozygous pathogenic variants in *GYG1*. Additionally, the patient was found to carry a variant of uncertain significance (VUS) in *RYR1* gene, known for its association with malignant hyperthermia and rhabdomyolysis (MIM 145600), which introduces additional complexity to this case.

This case highlights the diagnostic and clinical complexities of *GYG1*-related myopathy, underscoring the variability in presentation and progression of this rare disorder.

## Case report

**FIGURE 1:**
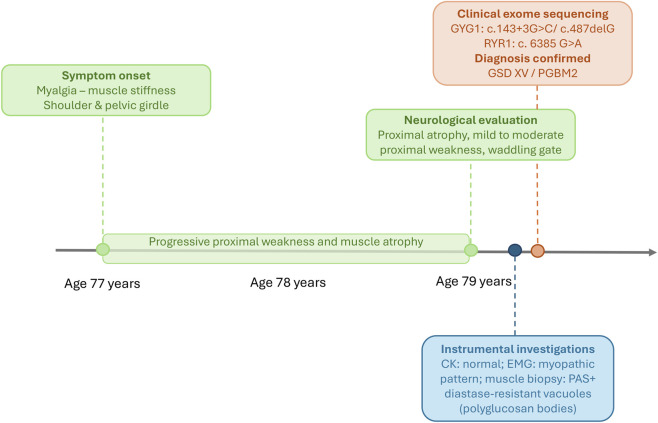
Main clinical events in patient’s clinical history.

Main clinical events in patient’s clinical history are available in Patient’s timeline (Figure 1). A 79-year-old Italian woman presented with progressive muscle stiffness and pain, primarily affecting the shoulder girdle, that had begun approximately 25 months earlier, when the patient was 77 years old. Over the time, she developed significant proximal weakness, more pronounced in the upper limbs, and progressive muscle atrophy. She reported increasing difficulty in raising her arms above her head, climbing stairs and performing fine motor tasks. Additionally, she experienced, occasional exertional dyspnea. The patient denied a history of statin use. Current medications included Vitamin D, ferrous sulphate, and folic acid. Past medical history was significant for osteopenia, colon carcinoma treated with surgery alone in 2004, pericarditis treated with pericardiocentesis in 2002, and a previous multifactorial anemia. The patient had received her last COVID-19 vaccine dose in December 2021, with symptom onset reported in late December 2021/early January 2022, in close temporal proximity to vaccination. No other routine vaccinations were administered within a clinically relevant timeframe prior to symptom onset. No family history of neuromuscular disease was reported, and parental consanguinity was denied by the patient.

Physical examination revealed severe muscle atrophy, predominantly in the proximal upper limbs, limited shoulder abduction (up to 30° on the right and 45° on the left), reduced strength graded as 3/5 Medical Research Council (MRC) scale for finger abductors and pinch strength bilaterally, symmetric mild proximal weakness (4/5 MRC) in the lower limbs. Reflexes were hypoactive in the upper limbs and normal in the lower limbs. She also demonstrated a waddling gait and scoliosis.

Electromyography (EMG) findings showed reduced amplitude motor potentials with early recruitment suggestive of a myopathic pattern more marked in the proximal muscles of the upper limbs. Laboratory results, including creatine kinase (CK) levels, complete blood count, hepatic and renal function, thyroid function, lactate, and inflammatory markers, were within normal ranges. Myositis-specific antibodies panel was negative. Twelve-lead ECG showed sinus rhythm, left anterior fascicular block, right bundle branch block, normal PQ interval, and non-specific repolarization abnormalities. Additional investigations included a thoracic CT scan, which revealed no pulmonary nodules or infiltrates, spirometry and nocturnal pulse oximetry which were within normal limits.

**FIGURE 2:**
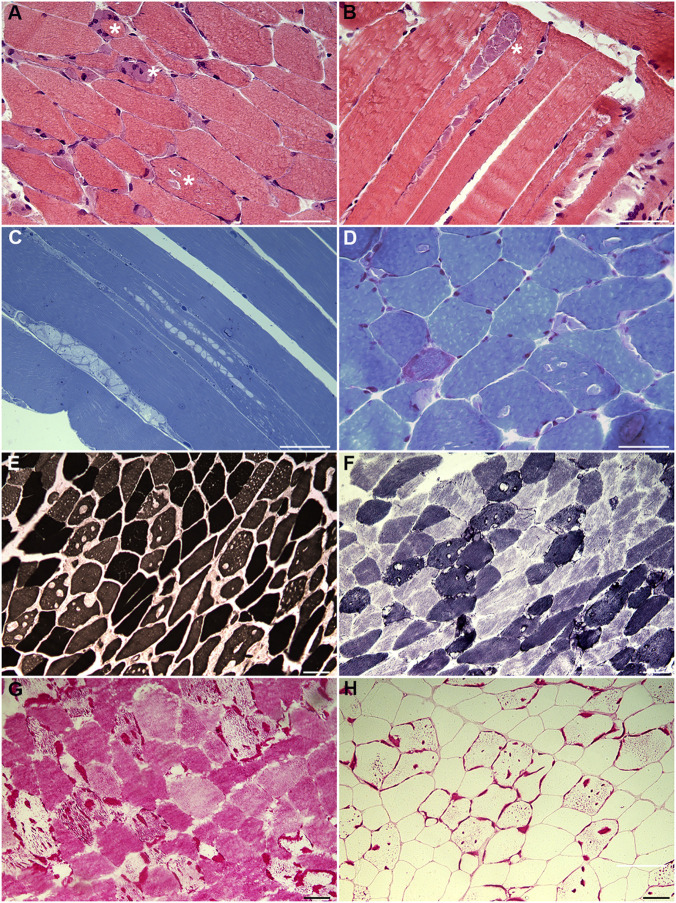
Morphological findings. H&E staining **(A,B)** and semithin section **(C)** show the presence and the distribution of vacuoles in muscle fibers, some necrotic fibers being also evident [MGT staining, **(D)**]. ATPase staining at pH 9.4 **(E)** identifies vacuolated fibers as type I fibers. The enzymatic activity is non-homogeneously distributed [NADH staining, **(F)**]. Vacuoles show marked PAS-positivity **(G)**, confirmed after digestion with PAS-diastase **(H)**. Scale bar 20 µm.

Muscle biopsy of the left biceps brachii showed several fibers containing single or multiple vacuoles of varying size, filled with amorphous material and located at both intracytoplasmic and subsarcolemmal level. The examination of semithin sections allowed us to better characterize both morphologic features and location of the vacuoles (Figures 2A–C). Necrotic and degenerating fibers were also observed along with scattered hypotrophic fibers (Figure 2D). ATPase staining revealed that vacuolated fibers correspond to type 1 fibers (Figure 2E). Oxidative enzymatic activity was unevenly distributed, the activity being increased in the hypotrophic fibers and absent within the vacuoles (Figure 2F). Additionally, Cytochrome c Oxidase (COX) activity was reduced in few fibers. The vacuoles displayed a strong PAS-positivity, which persisted, mostly as bigger dotty inclusions, after PAS-diastase digestion (Figures 2G,H).

**FIGURE 3:**
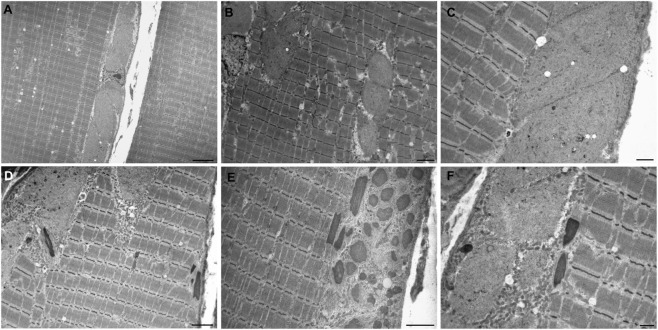
Ultrastructural studies. Numerous polyglucosan bodies distributed in both subsarcolemmal and intrafibrillar regions resembling a grape-like organization **(A–C)**. Polyglucosan bodies at high magnification, revealing a filamentous structure interspersed with sparse glycogen granules **(C)**. **(D–F)**: Nemaline bodies, observed either as isolated structures **(D,F)** or clustered **(E)** predominantly in subsarcolemmal regions. Sarcomeres showing jagged Z-lines **(F)**. Scale bars: **(A)** 5 μm; **(B–E)** 2.5 μm; **(F)** 1 µm.

Ultrastructural analysis revealed the presence of numerous polyglucosan bodies distributed in both subsarcolemmal and intrafibral regions (Figures 3A,B). These structures significantly vary in size, with some reaching considerable lengths of up to 20–30 sarcomeres. Morphologically, they exhibit a distinctive lobulated appearance, resembling a grape-like organization. At high magnification, they appear to be composed of filamentous material interspersed with sparse glycogen granules (Figure 2C). Notably, multiple polyglucosan bodies were often observed within the same fiber. Moreover, a considerable number of sarcomeres exhibit a jagged Z-line (Figure 3F), indicating alterations in the structural integrity of the contractile apparatus. Additionally, some fibers display nemaline bodies (Figures 3D–F), which were observed either as isolated structures or clustered predominantly in subsarcolemmal regions. Interestingly, nemaline bodies were occasionally found adjacent to polyglucosan bodies, suggesting a potential spatial relationship between these structures.

Clinical exome sequencing revealed the presence of two variants in *GYG1*, both classified as pathogenic according to ACMG criteria: the c.143 + 3G>C variant (intron 2, ClinVar ID 162661) and the c.487delG, p. (Asp163Thrfs*5) variant (exon 5, ClinVar ID 162665). Both the variants were previously published (Malfatti et al., 2014; Desikan et al., 2018; Akman et al., 2016). These findings, together with the patient’s clinical presentation, confirmed a diagnosis of PGBM2 caused by molecular defects in the *GYG1* gene.

Molecular analysis also identified the heterozygous c.6385G>A, p. (Asp2129Asn) variant in the *RYR1* gene (ClinVarID 570420), classified as likely pathogenic according to ACMG criteria and associated with exertional myalgia and rhabdomyolysis (9, MIM 145600).

Segregation analysis of the identified variants was not available due to the unavailability of parental DNA and the refusal of the proband’s daughters to undergo genetic testing.

All instrumental investigations and genetic testing were performed at 79 years of age, during the same clinical workup initiated at our centre.

At the latest neurological evaluation, performed 6 months after diagnosis, the patient exhibited a stable neuromuscular condition, with ubiquitously reduced muscle trophism and more evident weakness in the upper limbs. There were no speech disturbances, dysphagia, dyspnoea, or alterations in tactile sensitivity. Follow-up investigations, including cardiac monitoring, creatine kinase levels and a family genetic study, were recommended.

## Discussion and conclusion

Clinical presentation of *GYG1*-related myopathy is highly variable, ranging from mild, slowly progressive muscular weakness to severe neuromuscular involvement leading to wheelchair-dependency. Most patients present with limb-girdle weakness, while some exhibit scapuloperoneal, distal or asymmetric patterns (Malfatti et al., 2014; Ben Yaou et al., 2017; Stojkovic et al., 2018; Desikan et al., 2018; Akman et al., 2016; Lefeuvre et al., 2020; Colombo et al., 2016). Our patient’s phenotype aligns with the predominant proximal weakness reported in current studies. In addition to skeletal myopathy, some patients present with a more extensive disease, including cardiomyopathy and respiratory involvement: cases of cardiomyopathy requiring transplantation have been also reported (Moslemi et al., 2010; Hedberg-Oldfors et al., 2017). Our patient demonstrated no cardiac or respiratory compromise, suggesting a milder disease course, however, reports of cardiomyopathy underline the importance of ongoing cardiac surveillance, even in asymptomatic patients. Notably, her advanced age at diagnosis underscores the potential for late-onset manifestations, which, although documented, remain underrepresented in the literature (Tasca et al., 2016).

The *GYG1* variants identified in this patient, c.143 + 3G>C and c.487delG, are well-documented pathogenic variants, previously reported in 23 and 7 patients, respectively (Malfatti et al., 2014; Desikan et al., 2018; Akman et al., 2016). While these variants are frequently found in homozygosis, their co-occurrence in a patient constitutes a unique genotype.

To disclose a genotype-phenotype correlation, we conducted a comprehensive analysis of 44 patients with confirmed pathogenic variants reported in the literature (Supplementary Table 1). Most of the patients harbour biallelic null molecular defects (n = 32) including frameshift, nonsense, or splice-site variants in homozygosis or compound heterozygosis (Supplementary Table 2). In this group, a predominantly proximal or proximal-distal involvement of the muscles is observed with a highly variable age of presentation and cardiac involvement in approximately one-third of the cases. The most frequent variant in this group is c.143 + 3G>C p. (Asp3Glufs4), which disrupts RNA splicing, resulting in reduced glycogenin protein levels. Other nonsense mutations, such as c.484delG and c.970C>T, have been reported across individuals from various countries (Malfatti et al., 2014; Ben Yaou et al., 2017). These defects lead to a truncated protein that lacks the crucial C-terminal domain and abolish glycogenin-1 activity. The resulting protein is incapable of self-glycosylation leading to severe reduction in glycogen synthesis and increased accumulation of polyglucan bodies.

Patients with homozygous missense mutations present a clinical profile characterized by higher rate of cardiac involvement (3 out of 4 cases), with no or mild skeletal muscle involvement. These mutations, although impairing catalytic activity, preserve partial protein expression in skeletal muscle. The c.304G>C, p. (Asp102His) mutation accounts for most cases in this category and appears to confer increased risk of heart disease requiring heart transplantation in some cases.

Patients with compound heterozygous mutations combining missense and null variants demonstrate a low rate of cardiac involvement and predominant proximal muscular weakness, although variable distributions including scapuloperoneal and distal presentations have been reported.

Our patient presents a unique compound heterozygous combination of c.143 + 3G>C (splice-site) and c.487delG (frameshift), both representing loss-of-function mutations. She had a later onset and a muscle-restricted phenotype, which aligns her with the predominantly myopathic, later-onset presentation typically observed in patients carrying compound heterozygous mutations and supporting the hypothesis that specific allelic combinations can modulate clinical severity.

Genotype-phenotype correlation could have implications in clinical management, in defining the best follow-up strategy and in offering more accurate prognostic counselling based on the specific mutational profile of individual patients. However, phenotypic variability among patients with similar genotypes underscores the role of additional genetic, epigenetic, or environmental modifiers (Malfatti et al., 2014; Ben Yaou et al., 2017; Stojkovic et al., 2018; Desikan et al., 2018; Akman et al., 2016; Lefeuvre et al., 2020; Colombo et al., 2016; Moslemi et al., 2010; Hedberg-Oldfors et al., 2017; Tasca et al., 2016; Laforêt et al., 2017). As an example, the presence of the *RYR1* variant p. (Asp2129Asn) in this case report may further contribute to clinical presentation and ultrastructural findings.

*RYR1* gene encodes the ryanodine receptor 1, a calcium release channel essential for excitation-contraction coupling in skeletal muscle. Molecular defects in *RYR1* are associated with a spectrum of muscle disorders, ranging from congenital myopathies to episodic conditions like Malignant Hyperthermia Susceptibility (MHS) and Exertional Rhabdomyolysis. These mutations can disrupt calcium homeostasis in skeletal muscle, potentially increasing susceptibility to metabolic or stress-related triggers (Lawal et al., 2020; Snoeck et al., 2015; Dlamini et al. 2013). Although previously associated with exertional rhabdomyolysis in a dominant inheritance pattern, the pathogenetic role of the *RYR1* variant p. (Asp2129Asn) is still unclear. It is important to emphasize that this variant is classified as likely pathogenic according to ACMG criteria, and its potential contribution to the clinical picture should not be dismissed, even in the absence of overt *RYR1*-related phenotypic features. Our patient did not exhibit clinical features commonly associated with *RYR1*-related disorders, the co-occurrence of *GYG1* mutations and the *RYR1* likely pathogenic variant may contribute to modulate disease severity. Functional studies could help determine whether the variant exacerbates muscle pathology through disrupted calcium homeostasis. While the variant has not been linked to significant complications in our patient, its presence may warrant caution in specific situations, such as exposure to triggering agents like volatile anaesthetics.

From a histopathological point of view, while the presence of polyglucosan bodies is a hallmark of *GYG1*-related myopathy, certain ultrastructural findings, particularly the Z-line abnormalities, are more commonly observed with congenital myopathies linked to *RYR1* molecular defects. Although the hallmark features of *RYR1*-related myopathies such as central cores, multi-minicores, or disorganized myofibrillar patterns were not clear in this patient, the observed Z-line irregularities (Figure 2F) may indicate subtle structural defects related to RYR1 dysfunction.

Symptom onset occurred in close temporal proximity to the patient’s last COVID-19 vaccine dose (December 2021), which may represent a coincidental finding. However, we cannot entirely exclude the possibility that an immune-mediated event contributed to unmasking or exacerbating an underlying subclinical myopathy. This temporal association is noteworthy yet must be interpreted with caution: the patient’s overall clinical course is consistent with the natural history of GSD XV/PGBM2, and no immunological data supporting a causal relationship are available. The delayed presentation precluded the evaluation of early CK levels, which could have provided additional context. This hypothesis remains speculative and should not be overinterpreted. Further studies are needed to better define genotype-phenotype correlations in PGBM2 and to explore the impact of potential modifier genes and the interplay between multiple genetic variants on disease expression.

## Data Availability

The original contributions presented in the study are included in the article/Supplementary Material, further inquiries can be directed to the corresponding author.
