# Supplementary material for: Glycogenin-1 deficiency: a case report and review of the literature
Source: Front Genet. 2026 May 29;17:1777448. doi: 10.3389/fgene.2026.1777448 (PMC13258228; doi:10.3389/fgene.2026.1777448)
Supplement: Supplementary file 1 [file DataSheet1.pdf]

# Patient	# Family	Reference PMID	ID	Gender	Allele 1 [ClinVar ID]	Allele 2 [ClinVar ID]	Age Onset	Age Last Examination	Clinical Findings	Muscle Involvement	Respiratory Involvement	Cardiac involvement	Ambulation status	Additional clinical aspects	EMG	CK	Pas+ vacuoles	Muscle biopsy: additional findings
1	1	20357282	Patient	M	c.487del, p.(Asp163Thrfs*5) [162665]	c.248C>T, p.(Thr83Met) [5954]	childhood	27	Muscle weakness	Scapulo-peroneal (Hips and tights not involved)	Yes (mild)	cardiac arrhythmia (ventricular fibrillation)	Ambulant	Scapular winging	Myopathic	N	Y (heart), N (sk.- muscle)	Heart: enlarged cardiomyocytes. Muscle: type 1 predominance, mitochondrial proliferation
2	2	25272951	P1	M	c.143+3G>C, p.(Asp3Glufs*4) [162661]	c.143+3G>C p.(Asp3Glufs*4) [162661]	17	26	Muscle weakness, atrophy, fatigability	Proximal	No	Normal	Ambulant		Myopathic	I	Y	Inclusions (Ub, p62, desmin). Increased connective tissue (P3, P5)
3	3		P2	F	c.143+3G>C, p.(Asp3Glufs*4) [162661]	c.143+3G>C p.(Asp3Glufs*4) [162661]	childhood	50	Muscle weakness	Proximal to Distal	No	Normal	Ambulant		Myopathic	N	Y	
4	4		P3	M	c.304G>C, p.(Asp102His) [162663]	c.749G>A p.(Trp250*) [162664]	39	43	Muscle weakness	Proximal to Distal	No	Normal	Ambulant		Myopathic	N	Y	
5	5		P4	F	c.46G>C, p.(Ala16Pro) [NA]	c.46G>C, p.(Ala16Pro) [NA]	65	72	Muscle weakness	Proximal	No	Normal	Ambulant		Myopathic	N	Y	
6	6		P5	M	c.143+3G>C, (p.Asp3Glufs*4) [162661]	c.7G>C, spl. [NA]	62	72	Muscle weakness	Proximal	No	Normal	Ambulant	Asymmetric involvement of the arms	Mixed	N	Y	
7	7		P6	F	c.487del, p.(Asp163Thrfs*5) [162665]	c.487del, p.(Asp163Thrfs*5) [162665]	61	66	Muscle weakness	Distal	No	Normal	Ambulant		Myopathic	N	Y	
8	8		P7	F	c.143+3G>C, (p.Asp3Glufs*4) [162661]	c.970C>T, p.(Arg324*) [162662]	49	57	Muscle weakness	Proximal to Distal	No	Normal	Ambulant		Myopathic	N	Y	
9	9	26255073	Patient	F	c.634C>T, p.(His212Tyr) [NA]	c.634C>T, p.(His212Tyr) [NA]	39	46	Muscle weakness, atrophy, fatigability	Proximal	No	Normal	Ambulant	Asymmetric weakness, scapular winging, bilateral neurosensory hearing loss	Myopathic	N	Y	Type 1 fiber predominance, inclusions (desmin)
10	10		Patient 1	M	c.143+3G>C, p.(Asp3Glufs*4) [162661]	c.143+3G>C, p.(Asp3Glufs*4) [162661]	adult	74	Muscle weakness	Proximal to Distal	No	conduction block, ischemic cardiomyopathy	Ambulant	Vitiligo, calf hypertrophy, pes cavus		I	No	Myopathic features
11	10		Patient 2 (sibling)	F	c.143+3G>C, p.(Asp3Glufs*4) [162661]	c.143+3G>C, p.(Asp3Glufs*4) [162661]	52	60	Muscle weakness, hand muscle atrophy	Proximal to Distal	No	Normal	Unable to walk unsupported at 60 years	HSP, CIDP	Normal	I	Y	Inclusions (desmin, Ub).
12	10		Patient 3 (cousin)	F	c.143+3G>C, p.(Asp3Glufs*4) [162661]	g.148717967C>G, spl., p.? [NA]	50	70	Muscle weakness myalgia, atrophy	Proximal	No	Normal	Ambulant	Scapular winging		N	Y	1st biopsy: myopathic features; 2nd biopsy: inclusions (desmin, Ub).
13	11	26652229	P1	M	c.143+3G>C, p.(Asp3Glufs*4) [162661]	c.143+3G>C, p.(Asp3Glufs*4) [162661]	40	63	Muscle weakness, myalgia, exercise intolerance	Proximal	No	ischemic cardiomyopathy	Ambulant	Hyperlordosis	Mixed	N	Y	
14	12		P2	M	c.143+3G>C, p.(Asp3Glufs*4) [162661]	c.143+3G>C, p.(Asp3Glufs*4) [162661]	53	65	Muscle weakness	Proximal	No	mild (dilation of aortic root; slight left atrial dilation)	Ambulant	Scapular winging	Neurogenic (radiculopathy)	I	Y	
15	13		P3	F	c.143+3G>C, p.(Asp3Glufs*4) [162661]	c.143+3G>C, p.(Asp3Glufs*4) [162661]	55	77	Muscle weakness	Proximal	No	mild (sclerosis of aortic valve, slight mitral and tricuspid insufficiency)	Ambulant	Kiphosis	Myopathic	N	Y	
16	14		P4	F	c.143+3G>C, p.(Asp3Glufs*4) [162661]	c.143+3G>C, p.(Asp3Glufs*4) [162661]	60	80	Muscle weakness	Proximal	No	mild (hypertensive cardiomyopathy)	Ambulant		Myopathic	N	Y	
17	14		P5	F	c.143+3G>C, p.(Asp3Glufs*4) [162661]	c.143+3G>C, p.(Asp3Glufs*4) [162661]	45	65	Muscle weakness, exercise intolerance	Proximal	No	mild (slight mitral and tricuspid insufficiency)	Ambulant		Myopathic	N	No	
18	15	27544502	Patient	M	c.2T>A, p.? [1508529]	c.2T>A, p.? [1508529]	82	84	Muscle weakness	Proximal to Distal	No	mild (BAV I)	Ambulant	Asymmetric weakness in LL, bilateral neurosensory hearing loss	Myopathic	N	Y	Inclusions (desmin, p62). Nemaline rods (EM)
19	16	29143313	Patient 1	F	c.403G>A, p.(Gly135Arg) [1381640]	c.487del, p.(Asp163Thrfs*5) [162665]	40	66	Muscle weakness	Distal	No	Normal	Ambulant		Myopathic	N	Y	Inclusions (desmin, p62)
20	16		Patient 2 (sibling)	M	c.403G>A, p.(Gly135Arg) [1381640]	c.487del, p.(Asp163Thrfs*5) [162665]	37	64	Muscle weakness. Atrophy.	Proximal	No	hypertrophic cardiomyopathy with predominant left ventricular hypertrophy, FAP	Ambulant	Asymmetric weakness and atrophy at LL	Myopathic	I	Y	
21	17	28453664	Patient 1	F	c.166G>C, p.(Asp56His) [NA]	c.472del, p.(Ser158Valfs*10) [NA]	50	77			No	Normal	Ambulant				Y	Inclusions (desmin, filamin C)
22	18		Patient 2	F	c.487del, p.(Asp163Thrfs*5) [162665]	c.487del, p.(Asp163Thrfs*5) [162665]	30	46			No	Normal	Ambulant				Y	
23	19	29205400	Patient	F	c.143+3G>C, p.(Asp3Glufs*4) [162661]	c.143+3G>C (p.Asp3Glufs*4) [162661]	46	63	Muscle weakness	Proximal to Distal	No	Normal	Ambulant	Asymmetric deltoid weakness, scapular winging	Myopathic	N	Y	Inclusions (desmin, TDP43)
24	20	27718144	Patient 1	M	c.304G>C, p.(Asp102His) [162663]	c.304G>C, p.(Asp102His) [162663]	34	49	Myalgia	No	No	severe cardiac hypertrophy arrhythmia, heart	Ambulant			I	Y (muscle and heart)	

												transplantation at 48 years						
25	21		Patient 2	M	c.304G>C, p.(Asp102His) [162663]	c.304G>C, p.(Asp102His) [162663]	46	52		No	No	severe cardiac hypertrophy arrhythmia, heart transplantation at 52 years	Ambulant		Normal	N	Y (heart)	
26	22		Patient 3	M	c.304G>C, p.(Asp102His) [162663]	c.304G>C, p.(Asp102His) [162663]	23	34		No	No	severe cardiac hypertrophy arrhythmia, ICD at 31 years	Ambulant			N	Y (heart)	
27	23		A-1	M	c.143+3G>C, p.(Asp3Glufs*4) [162661]	c.646C>T, p.(Arg216*) [1879594]	28	32	Muscle weakness. Myalgia.	Scapulo-peroneal	Yes (mild)	Normal	Ambulant	Asymmetric scapular weakness	Myopathic	I	Y	
28	24		B-1	M	c.143+3G>C, p.(Asp3Glufs*4) [162661]	c.143+3G>C, p.(Asp3Glufs*4) [162661]	16	27	Muscle weakness	Proxymal	No	Normal	Ambulant	Scapular winging, calf ipertrophy	Myopathic	I	Y	
29	24		B-2	M	c.143+3G>C, p.(Asp3Glufs*4) [162661]	c.143+3G>C, p.(Asp3Glufs*4) [162661]	15	27	Muscle weakness, atrophy, fatigability	Proxymal	No	Normal	Ambulant	Scapular winging		I	Y	
30	24		B-3,	M	c.143+3G>C, p.(Asp3Glufs*4) [162661]	c.143+3G>C, p.(Asp3Glufs*4) [162661]	asymptomatic	23	Asymptomatyc		No	Normal	Ambulant			N		
31	25	29264399	C-1	F	c.143+3G>C, p.(Asp3Glufs*4) [162661]	c.143+3G>C, p.(Asp3Glufs*4) [162661]	47	58	Muscle weakness	Distal	Yes (OSAS requiring NIV)	AVB	Ambulant	Asymmetric distal LL (right than left)	Myopathic		N vacuolar myopathy	Mild necrosis, endomisial fibrosis, mitochondrial accumulation
32	25		C-2	M	c.143+3G>C, p.(Asp3Glufs*4) [162661]	c.996_1005del, p.(Tyr332*) [4084914]	72	72	Muscle weakness	Distal	No	Normal	Ambulant	Asymmetric distal LL (right than left)	Myopathic	N	Y	Mild endomysial fibrosis
33	25		C-3	F	c.143+3G>C, p.(Asp3Glufs*4) [162661]	c.996_1005del, p.(Tyr332*) [4084914]	79	81	Muscle weakness	Distal	No	Myocardial infarction at 76 y old requiring stenting	Ambulant		NP			Fatty replacement, myofibrillar disorganization
34	26		D-1	F	c.166G>C, p.(Asp56His) [NA]	c.472del, p.(Ser158Valfs*10) [NA]	50	76	Muscle weakness	Proximal	No	Normal	Ambulant	Asymmetric involvement	Myopathic	I	Y	
35	27		E-1	F	c.487del, p.(Asp163Thrfs*5) [162665]	c.487del, p.(Asp163Thrfs*5) [162665]	teen	45	Muscle weakness	Proximal	No	Normal	Ambulant		Myopathic	I	Y	
36	28	29422440	Patient	F	c.487del, p.(Asp163Thrfs*5) [162665]	c.487del, p.(Asp163Thrfs*5) [162665]	teen	84	Muscle weakness, atrophy, exertional myalgia	Proximal to Distal	No	Normal	Ambulant	Asymmetric weakness, asymmetric scapular winging, right side ptosis and mild tongue weakness	Myopathic	I	Y	Fatty replacement, selective type 2 fiber atrophy.
37	29	31791869	Patient 1	M	c.144–2A>G, spl. [NA]	c.631del, p.(Val211Cysfs*29) [855451]	53	70	Myalgia, cramps, muscle atrophy	Distal to Proximal	No	coronary artery disease	Ambulant	Scapular winging	Myopathic	I	Y	Type 1 fiber predominance, nuclear centralization, inclusions (desmin, α-actinin, titin).
38	30		Patient 2	M	c.304G>C, p.(Asp102His) [162663]	c.487del, p.(Asp163Thrfs*5) [162665]	70	77	Muscle weakness and atrophy	Proximal	No	Normal	Ambulant		Myopathic	N	Y	Mild endomysial fibrosis, type 1 muscle fiber predominance, nuclear centralizations, inclusions (myophosphorylase, p62)
39	31	32419263	Patient 13	M	c.164_165delTT, p.(Phe55*) [NA]	c.646C>T, p.(Arg216*) [1879594]	49	58	Muscle weakness	Distal > Proximal	No	Normal	Ambulant	Foot drop		I	Y	Myofibrillar disorganization, myonuclear lobulation, sarcoplasmic reticulum lobulation
40	32	32905144	Patient	F	c.143+3G>C, p.(Asp3Glufs*4) [162661]	c.819T>A, p.(Tyr273*) [850059]	58	68	Muscle weakness	Distal	No	Normal	Ambulant		Myopathic	N	Y	
41	33	32477874	Patient	M	c.304G>C, p.(Asp102His) [162663]	c.164_165delTT, p.(Phe55*) [NA]	25	44	Muscle weakness and atrophy. Myalgia	Proximal	No	Normal	Ambulant	Asymmetric weakness, scapular winging	Myopathic	I	Y	
42	34	34602496	Patient	F	c.7+992T>G, spl. [NA]	c.7+992T>G, spl. [NA]	teen	33	Muscle weakness	Proximal						I	Y	
43	35	26203156	P.IV-5	F	c.143+3G>C, p.(Asp3Glufs*4) [162661]	c.143+3G>C, p.(Asp3Glufs*4) [162661]	30	71	Muscle weakness	Proximal > Distal	No	Normal	Wheelchair at 59 years	Facial weakness	Myopathic	N	Y	
44	35		P.IV-10	F	c.143+3G>C, p.(Asp3Glufs*4) [162661]	c.143+3G>C, p.(Asp3Glufs*4) [162661]	53	64	Muscle weakness	Proximal	No	Normal	Ambulant	Scapular winging		N	Y	
45	36	This Study	Patient	F	c.143+3G>C, (p.Asp3Glufs*4) [162661]	c.487del, p.(Asp163Thrfs*5) [162665]	79	79	Muscle weakness, muscle atrophy	Proximal to Distal	No	(EAS + BBDA)	Ambulant	Scoliosis	Myopathic	N	Y	

Supplementary Table 1 Clinical, histological and molecular findings of previously reported *GYG1*-mutated patients.
Legend N: normal; I: increased; EMG: electromyography; CK: serum creatine kinase levels; NA: not available. the following terms were searched through PubMed in December 2025, filtering for human studies, abstract and full-text availability in English: "(GYG1) AND (polyglucosan body)) OR (glycogen storage disease)." We included publications reporting patients of any age, and providing clinical, instrumental, and molecular characterization, with the latter clearly specifying the presence of GYG1 biallelic variants, either carried in homozygosis or compound heterozygosis.

Genotype	n (%)	Age of Onset		Cardiac Involvement (%)	Most Common Muscle Pattern	Representative variants [ACMG, ClinVar ID]
		Mean (years)	Range (years)			
**Biallelic Null (LoF)**<br>Frameshift, nonsense, splice-site	32 (73%)	43.7	10-82	31.3%	Proximal (43.8%)<br>Proximal-to-distal (28.1%)	• c.143+3G>C p.(Asp3Glufs*4) [P, 162661] (n=12)<br>• c.487del, p.(Asp163Thrfs*5) [P, 162665] (n=4)<br>• c.996_1005del, p.(Tyr32*) [LP, 4084914] (n=2)
**Homozygous Missense**	4 (9%)	42	23-65	75.0%	Proximal (25%)<br>Cardiac-predominant (75%)	• c.304G>C, p.(Asp102His) [LP, 162663] (n=3)<br>• c.46G>C, p.(Ala16Pro) [VUS, NA] (n=1)
**Compound Heterozygous**<br>Missense + Null	8 (18%)	40.1	10-70	25.0%	Proximal (50%)<br>Variable distribution	• c.403G>A [VUS, 1381640] + c.487del [P, 162665] (n=2)<br>• c.166G>C [VUS, NA] + c.472del [LP, NA] (n=2)<br>• c.304G>C [LP, 162663] + c.487del [P, 162665] (n=1)
**Present Case**<br>Compound Heterozygous	1	79	79	0%	Proximal	c.143+3G>C [P, 162661] + c.487delG [P, 162665]

Supplementary Table 2 Genotype-Phenotype Correlation in GYG1-Related Myopathy: Analysis of 44 Patients from Literature Review

Legend and Clinical Implications

- Biallelic Null variants: greatest phenotypic variability; moderate cardiac risk; require comprehensive screening.
- Homozygous Missense variants: highest cardiac involvement; require intensive cardiological monitoring.
- Compound Heterozygous variants: low rate of cardiac involvement; predominant proximal muscular weakness.
- Present Case: late onset; supports role of genetic/environmental modifiers

LoF: Loss-of-function; n: number of patients. ACMG classification (P: Pathogenic, LP: Likely Pathogenic, VUS: Variant of Uncertain Significance) and CliVar ID are indicated within square brackets
